# PCR array analysis identified hyperproliferation but not autophagy or apoptosis in fibrous epulis

**DOI:** 10.1002/jcla.23784

**Published:** 2021-05-02

**Authors:** Yangyang Jiang, Bing Fang, Bin Xu

**Affiliations:** ^1^ Department of Orthodontics Shanghai Ninth People’s Hospital Shanghai Jiao Tong University School of Medicine Shanghai China; ^2^ Department of Stomatology Hwa Mei Hospital University of Chinese Academy of Sciences Ningbo China

**Keywords:** apoptosis, ATG family, autophagy, Bcl‐2 family, caspase family, fibrous epulis, PCR array

## Abstract

**Background:**

The pathogenesis of fibrous epulis is still quite unclear. Our recent genome‐wide RNA sequencing analysis revealed that in fibrous epulis, RAS‐PI3K‐AKT‐NF‐κB pathway regulates the expression of Bcl‐2 family and IAP family genes, leading to increased proliferation and the inhibition of apoptosis. The PI3K/AKT signaling pathway can promote autophagy in human gingival fibroblasts; therefore, the purpose of the present study was to identify whether autophagy is involved in the pathogenesis of fibrous epulis.

**Methods:**

Differentially expressed genes (DEGs) between fibrous epulis lesions and normal gingival tissues were identified using the PCR array. The expression levels of eighteen autophagy‐related (ATG) family genes, twelve B‐cell lymphoma 2 (Bcl‐2) family genes, and eleven cysteine‐dependent aspartate‐directed protease (caspase) family genes were validated using quantitative real‐time PCR (qRT‐PCR). Autophagy induction was determined by measuring microtubule‐associated protein light chain 3 (LC3) conversion (LC3‐I to LC3‐II) by immunoblot analysis.

**Results:**

The PCR array identified six upregulated genes, whereas no genes were expressed at significantly lower levels. The upregulated genes were *BCL2*, *BCL2L1*, *CXCR4*, *HSP90AA1*, *HSPA8*, and *IGF1*, which all belong to the “regulation of autophagy” group but not the “autophagy machinery components” group. qRT‐PCR verified that the expression levels of *BCL2*, *BCL2L1* (also known as *BCL*‐*XL*), and *BCL2L2* (also known as *BCL*‐*W*) were significantly increased in fibrous epulis. No LC3‐I to LC3‐II conversion was observed.

**Conclusions:**

The present study reveals that in fibrous epulis, Bcl‐2 and Bcl‐xL coordinately mediate gingival cell escape from apoptosis, leading to uncontrolled proliferation. Moreover, ATG family genes are not activated, and autophagy is not involved in this process.

## Graphical abstract

The expression levels of eighteen autophagy‐related (ATG) family genes, twelve B‐cell lymphoma 2 (Bcl‐2) family genes, and eleven cysteine‐dependent aspartate‐directed protease (caspase) family genes between fibrous epulis lesions and normal gingival tissues were measured using quantitative real‐time PCR (qRT‐PCR). The result showed that the expression levels of *BCL2*, *BCL2L1* (also known as *BCL*‐*XL*), and *BCL2L2* (also known as *BCL*‐*W*) were significantly increased in fibrous epulis. The present study reveals that in fibrous epulis, Bcl‐2, and Bcl‐xL coordinately mediate gingival cell escape from apoptosis, leading to uncontrolled proliferation. Moreover, ATG family genes are not activated, and autophagy is not involved in this process.

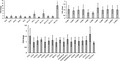

## 1 INTRODUCTION

The pathogenesis of fibrous epulis is still quite unclear, and apoptosis inhibition through the overexpression of Bcl‐2 may play an important role in the process of epulis.^1^, ^2^, ^3^ To investigate the molecular mechanisms of epulis, we recently used genome‐wide RNA sequencing (RNAseq) to identify alterations in gene expression in the transcriptome. RNAseq and subsequent KEGG pathway analysis indicated that the differentially expressed genes (DEGs) were enriched in “Ras signaling pathway” and “Pathways in cancer”.^4^ Moreover, the identified DEGs in epulis were not a part of the canonical RAS‐RAF pathway but in the RAS‐PI3K‐AKT pathway, which strongly indicated that although epulis has a tumor‐like appearance, it is not a true neoplasia because the critical signaling pathways were all associated with proliferation and antiapoptosis but not other cancer pathways.^4^ In another study, we identified the expression of 84 apoptotic genes in epulis using a Qiagen RT2 Profiler PCR Array, and the results indicated that the overexpression of antiapoptotic genes in the Bcl‐2 and IAP families inhibits apoptosis in gingival tissues, which eventually causes epulis.^5^ In summary, our recent studies revealed that in fibrous epulis, the RAS‐PI3K‐AKT‐NF‐κB pathway transcriptionally regulates the expression of Bcl‐2 family and IAP family genes, leading to increased proliferation and the inhibition of apoptosis.^4^, ^5^

A previous electron microscopic study of congenital epulis showed that the cells were filled with autophagic vacuoles that were devoid of normal cellular organelles.^6^, ^7^ A recent study also reported that the PI3K/AKT signaling pathway can promote autophagy in human gingival fibroblasts.^8^ Therefore, the present study used the PCR array to identify whether, in addition to increased proliferation and apoptosis inhibition, autophagy was involved in the pathogenesis of fibrous epulis. These results may provide novel information for further development of mechanism‐based diagnostics and therapies.

## 2 MATERIALS AND METHODS

### 2.1 Patient recruitment and sample collection

From January 2018 to December 2018, 55 patients with fibrous epulis were recruited for the current study from the Ninth People's Hospital affiliated with Shanghai Jiao Tong University, School of Medicine and Hwa Mei Hospital affiliated with the University of the Chinese Academy of Science. Fibrous epulis was diagnosed clinically and confirmed by histopathology, and the inclusion and exclusion criteria were described previously.^4^ Finally, five randomly selected patients, two males and three females, aged 43‐57 years, were recruited for PCR array analysis. Other 50 patients, 18 males and 32 females, aged 38‐61 years, were recruited for validation study. Each removed lesion and normal gingival tissue was divided into two pieces; one piece was stored in RNAstore for subsequent RNA extraction, and the other piece was stored in −80℃ refrigerator for subsequent immunoblot analysis. The protocol for this study was reviewed and approved by the Medical Ethics Review Board, and gingival tissues were collected with written informed consent.

### 2.2 RNA extraction

Total RNA was extracted from gingival tissues by using a Qiagen RNeasy Universal Tissue Kit with gDNA Eliminator Solution according to the manufacturer's instructions. The RNA quality was determined using a NanoDrop ND‐1000 spectrophotometer. RNA integrity was determined using an Agilent 2100 Bioanalyzer. The intensity of the 18S and 28S rRNA bands was examined on a 1% formaldehyde‐agarose gel. RNA samples with optical density (OD) 260/280 ratios ≥ 1.8, RNA integrity numbers (RINs) of ≥ 7.0 and 28S/18S > 1.5 were subjected to the following experiments.

### 2.3 PCR array analysis

The Qiagen RT^2^ Profiler PCR Array Human Autophagy was used to measure the expression levels of autophagy‐associated genes. This PCR array system is a reliable and accurate tool for analyzing the expression of 84 genes associated with autophagy machinery components (autophagic vacuole formation, vacuole targeting, protein transport, autophagosome‐lysosome linkage, ubiquitination, and proteases) and the regulation of autophagy (coregulators of autophagy and apoptosis, coregulators of autophagy and the cell cycle, autophagy induction by intracellular pathogens, autophagy in response to other intracellular signals, and chaperone‐mediated autophagy). Moreover, five housekeeping genes, one genomic DNA control, three reverse transcription controls, and three positive PCR controls were included in the same 96‐well plate.

The procedure began with the conversion of each 0.5 μg RNA sample into first‐strand cDNA using the RT^2^ First Strand Kit. Next, the cDNA was mixed with an appropriate RT^2^ SYBR^®^ Green Master mix. This mixture was aliquoted into the wells of the RT^2^ Profiler PCR Array. PCR was performed, and relative expression was determined using data from the real‐time cycler and the ΔΔCT method.

### 2.4 Quantitative reverse transcription‐Polymerase Chain Reaction (qRT‐PCR)

Eighteen autophagy‐related (ATG) family genes, twelve B‐cell lymphoma 2 (Bcl‐2) family genes, and eleven cysteine‐dependent aspartate‐directed protease (caspase) family genes were further validated by qRT‐PCR in 50 pairs of gingival tissues. Double‐stranded cDNA was synthesized using the TaKaRa PrimeScript RT Reagent Kit according to the manufacturer's instructions. Subsequently, qRT‐PCR was performed using a Roche LightCycler 480 and SYBR Green I Master mix. The ΔΔCT method was used to quantify the relative expression of each mRNA using GAPDH as an internal control. All the experiments were repeated three times.

### 2.5 Autophagy detection

Autophagy induction was determined by measuring microtubule‐associated protein light chain 3 (LC3) conversion (LC3‐I to LC3‐II) by immunoblot analysis, since this is currently the most experimentally straightforward method to monitor autophagic activity.^9^ Gingival tissues were homogenized in lysis buffer, and after being incubated on ice and centrifuged, the supernatant was carefully collected. Protein content was quantified by the Lowry method.^10^ Aliquots of supernatant containing 50 μg of protein were subjected to 10% SDS‐PAGE and transferred to a PVDF membrane. The membranes were blocked at room temperature for 2 h and then incubated with an anti‐LC3 primary antibody and an appropriate secondary antibody. The membranes were washed three times and exposed to ECL chemiluminescence reagents. The autoradiograms were scanned, and the protein bands were quantified by densitometry using Bio‐Rad Quantity One software.

### 2.6 Statistical analysis

The differences in mRNA expression between the groups were evaluated with paired‐samples t tests using SPSS 18.0 software. Significance was considered to be *p* < 0.05. The criteria for the PCR array were set as *p* < 0.05 and fold change ≥1.5 or ≤0.67.

## 3 RESULTS

### 3.1 Identification of differentially expressed autophagy‐associated genes between fibrous epulis lesions and normal gingival tissues

The expression profiles of 84 autophagy‐associated genes were determined using a PCR array. The whole results were listed in the Table S1. Compared with normal gingival tissues, the fibrous epulis lesions exhibited six upregulated genes, whereas no genes were expressed at significantly lower levels. The upregulated genes were *BCL2*, *BCL2L1*, *CXCR4*, *HSP90AA1*, *HSPA8*, and *IGF1* (Table 1), which all belong to the “regulation of autophagy” group but not the “autophagy machinery components” group. Moreover, none of the ATG family genes were found to have altered expression levels in fibrous epulis.

**TABLE 1:** PCR array analysis identified the DEGs of fibrous epulis

Gene symbol	Fold	95%CI	*p*‐value
*BCL2*	2.8708	1.96, 3.78	0.002084
*BCL2L1*	2.4894	1.61, 3.37	0.007523
*CXCR4*	2.4534	1.50, 3.41	0.022578
*HSP90AA1*	2.6539	1.90, 3.41	0.000496
*HSPA8*	2.2733	0.97, 3.58	0.013304
*IGF1*	2.2162	1.16, 3.27	0.039901

### 3.2 Identification of ATG family gene expression in fibrous epulis lesions and normal gingival tissues

**FIGURE 1:**
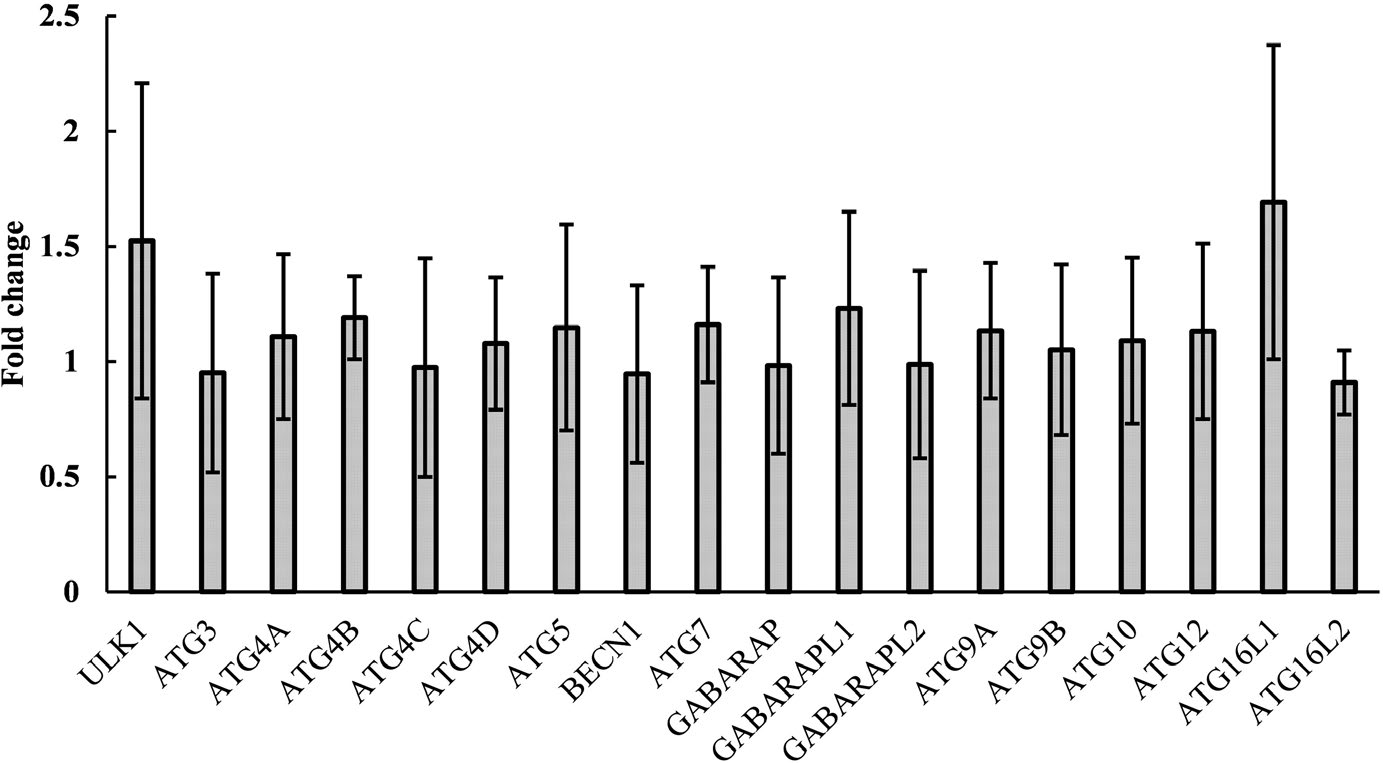
The expression levels of ATG family genes. The results are expressed as the mean ± SD from three independent experiments

Since no ATG family genes were identified in the PCR array, the expression levels of 18 ATG family genes were further measured by qPCR. These genes were *ULK1* (also known as *ATG1*), *ATG3*, *ATG4A*, *ATG4B*, *ATG4C*, *ATG4D*, *ATG5*, *BECN1* (also known as *ATG6*), *ATG7*, *GABARAP* (also known as *ATG8A*), *GABARAPL1* (also known as *ATG8B*), *GABARAPL2* (also known as *ATG8C*), *ATG9A*, *ATG9B*, *ATG10*, *ATG12*, *ATG16L1*, and *ATG16L2*. The results confirmed that the expression levels of ATG family genes were unchanged in fibrous epulis (Figure 1).

### 3.3 Identification of Bcl‐2 family gene expression in fibrous epulis lesions and normal gingival tissues

**FIGURE 2:**
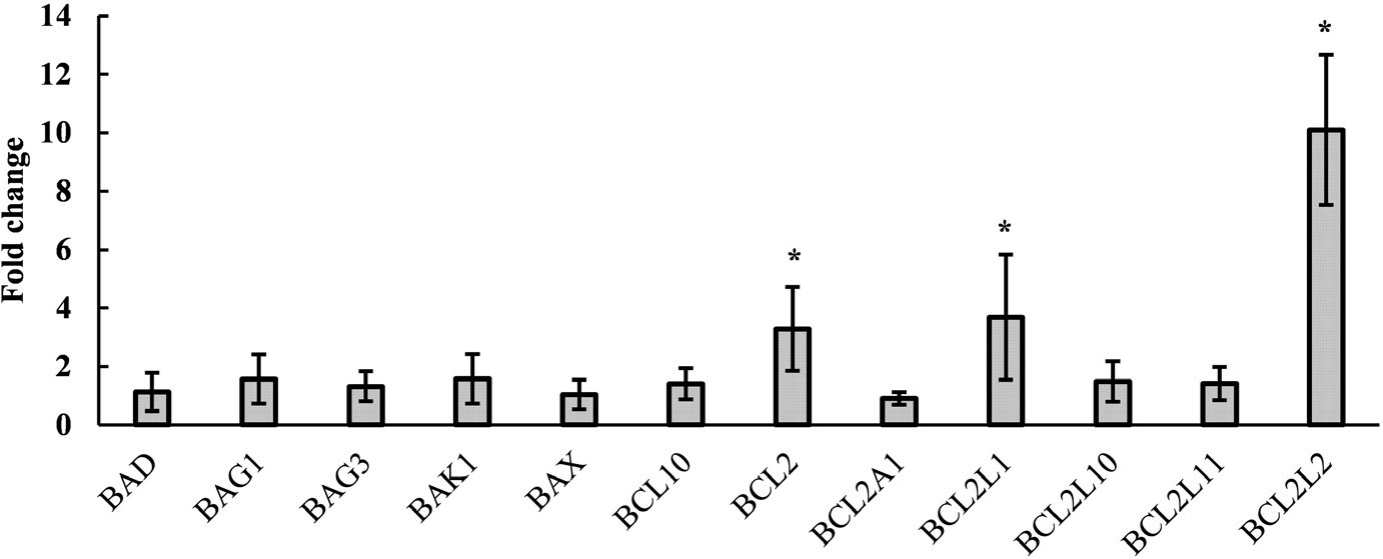
The expression levels of Bcl‐2 family genes. The results are expressed as the mean ± SD from three independent experiments. The asterisks represent statistical significance (*p* < 0.05) compared with the control group

The expression levels of 7 Bcl‐2 family genes were further measured by qRT‐PCR. These genes were *BCL2*, *BCL2A1*, *BCL2L1* (also known as *BCL*‐*XL*), *BCL2L10* (also known as *BCL*‐*B*), *BCL2L11* (also known as *BIM*), *BCL2L2* (also known as *BCL*‐*W*), and *BCL10*. The results indicated that the expression levels of *BCL2*, *BCL2L1*, and *BCL2L2* were significantly increased in fibrous epulis (Figure 2).

### 3.4 Identification of Caspase family gene expression in fibrous epulis lesions and normal gingival tissues

**FIGURE 3:**
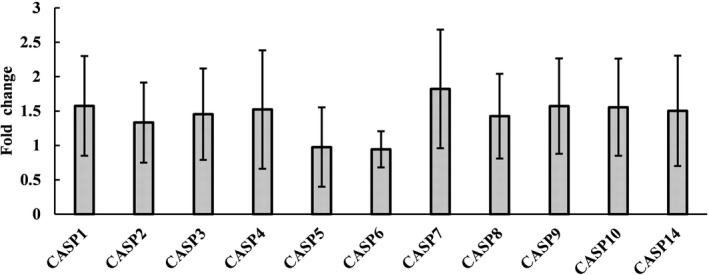
The expression levels of caspase family genes. The results are expressed as the mean ± SD from three independent experiments. The asterisks represent statistical significance (*p* < 0.05) compared with the control group

The expression levels of 11 caspase family genes were further measured by qRT‐PCR. These genes were *CASP1*, *CASP2*, *CASP3*, *CASP4*, *CASP5*, *CASP6*, *CASP7*, *CASP8*, *CASP9*, *CASP10*, *and* *CASP12*. The results indicated that the expression levels of all caspase family genes remained unchanged in fibrous epulis (Figure 3).

### 3.5 Identification of autophagy

**FIGURE 4:**
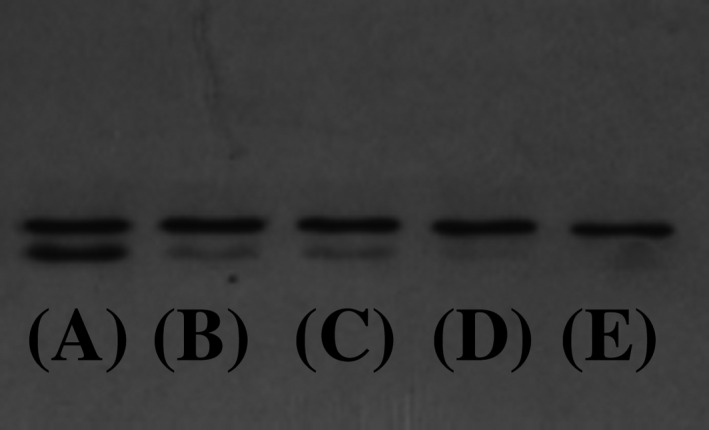
Identification of autophagy by LC3 conversion. The upper band is LC3‐I, and the lower band is LC3‐II. (A) is positive control; (B) is the normal gingival tissue from randomly selected sample X; (C) is the epulis lesion from randomly selected sample X; (D) is the normal gingival tissue from randomly selected sample Y; (E) is the epulis lesion from randomly selected sample Y

LC3 conversion was used to detect autophagy induction. According to the immunoblot analysis results, autophagy was not induced in fibrous epulis or normal gingival tissue (Figure 4). This result is consistent with the unchanged expression levels of ATG family genes.

## 4 DISCUSSION

Three major types of cell death occur under physiological conditions: apoptosis, autophagic cell death, and necrosis.^11^, ^12^ Apoptosis is usually characterized by a number of characteristic morphological changes, including cell shrinkage, chromatin condensation, nuclear fragmentation, and plasma membrane blebbing.^13^ It is widely known that three molecular mechanisms control apoptosis: the intrinsic pathway mediated by the Bcl‐2 family, the extrinsic pathway mediated by death receptors, and the endoplasmic reticulum pathway; all three pathways depend on caspase activation.^14^ Our previous RNAseq study and PCR array studies showed that the expression levels of *BCL2* and *BCL2L1* were significantly increased in fibrous epulis.^4^, ^5^ The BCL‐2 family, which includes both proapoptotic and antiapoptotic members, acts as a checkpoint upstream of caspase.^15^, ^16^ Therefore, in the present study, 12 Bcl‐2 family genes, including five proapoptotic members and seven antiapoptotic members, were investigated. The results indicated that all proapoptotic genes remained unchanged, while the expression levels of *BCL2*, *BCL2L1* (encoding the Bcl‐xL protein), and *BCL2L2* (encoding the Bcl‐w protein) were significantly increased in fibrous epulis. Interestingly, in the present autophagy PCR array analysis, *BCL2* and *BCL2L1* were also found to be increased significantly. Bcl‐2 and Bcl‐xL usually exhibit reciprocal expression patterns during development and carcinogenesis.^15^ Therefore, it is strongly suggested that in fibrous epulis, Bcl‐2 and Bcl‐xL coordinately mediate gingival cell escape from apoptosis, leading to uncontrolled proliferation.

Moreover, since the caspase cascade plays vital roles in the induction, transduction, and amplification of intracellular apoptotic signals,^17^ all caspase family genes were examined. The results indicated that none of the caspases were transcriptionally activated. Considering that the antiapoptotic members of the Bcl‐2 family and caspase members remained unchanged, it is quite clear that apoptosis is not involved in fibrous epulis but Bcl‐2‐mediated hyperproliferation is involved.

Our previous RNAseq study also showed that in fibrous epulis, apoptosis was inhibited through RAS‐PI3K‐AKT pathway‐mediated overexpression of Bcl‐2 family and IAP family genes. Moreover, a recent study also reported that the PI3K/AKT signaling pathway can promote autophagy in human gingival fibroblasts^8^; therefore, we conducted further analyses to identify whether autophagy was involved in fibrous epulis. Autophagy is a highly conserved cellular process that generally protects cells and organisms under various stress conditions; thus, autophagy has primarily cytoprotective functions and needs to be tightly regulated.^18^ In addition to its role in normal physiology, autophagy also plays a critical role in pathological processes such as cancer.^19^, ^20^ It is thought that autophagy prevents cancer development. Conversely, once cancer is established, increased autophagic flux often enables tumor cell survival and growth.^21^ Autophagy includes five phases: initiation, elongation, autophagosome formation, fusion, and autolysosome formation.^22^ During autophagy, the cytosolic form of LC3 (LC3‐I) is conjugated to phosphatidylethanolamine to form the LC3‐phosphatidylethanolamine conjugate (LC3‐II), which is recruited to autophagosomal membranes.^23^ Thus, measuring LC3 conversion (LC3‐I to LC3‐II) by immunoblotting has become a reliable method for monitoring autophagy and autophagy‐related processes. In the present study, LC3‐II was not obviously detected in epulis, indicating that autophagy may not be involved.

Autophagy is executed by ATG family genes.^24^ The ATG proteins involved in autophagosome formation consist of several functional units: the Atg1/ULK complex, the class III phosphatidylinositol 3‐kinase (PI3K) complex, the Atg2‐Atg18/WIPI4 complex, Atg9 vesicles, the Atg12 conjugation system, including ATG12–5‐16L1 and WIPI2B, and the Atg8/LC3 conjugation system.^25^, ^26^ Therefore, the expression levels of 18 ATG family genes were examined, and all examined genes remained unchanged in fibrous epulis. This result was consistent with the lack of LC3‐I to LC3‐II conversion, strongly indicating that similar to apoptosis, autophagy is not involved in the pathogenesis of fibrous epulis.

In conclusion, the present study reveals that in fibrous epulis, Bcl‐2 and Bcl‐xL coordinately mediate gingival cell escape from apoptosis, leading to uncontrolled proliferation. Moreover, ATG family genes are not activated, and autophagy is not involved.

## CONFLICTS OF INTERESTS

None.

## Supporting information

Table S1

## Data Availability

The data that support the findings of this study are available from the corresponding author upon reasonable request.

## References

[1] Seymour RA , Ellis JS , Thomason JM . Risk factors for drug‐induced gingival overgrowth. J Clin Periodontol. 2000;27(4):217‐223. 10783833 10.1034/j.1600-051x.2000.027004217.x

[2] Misra A , Rai S , Misra D . Functional role of apoptosis in oral diseases: an update. J Oral Maxillofac Pathol. 2016;20(3):491‐496. 27721616 10.4103/0973-029X.190953 PMC5051299

[3] Kuzenko Y , Romanyuk A , Politun A , Karpenko L . S100, bcl2 and myeloperoxid protein expirations during periodontal inflammation. BMC Oral Health. 2015;15:93. 26251029 10.1186/s12903-015-0077-8 PMC4527225

[4] Jiang Y , Fang B , Xu B , Chen L . The RAS‐PI3K‐AKT‐NF‐kappaB pathway transcriptionally regulates the expression of BCL2 family and IAP family genes and inhibits apoptosis in fibrous epulis. J Clin Lab Anal. 2020;34(3):e23102. 31743516 10.1002/jcla.23102 PMC7083487

[5] Jiang YY , Xu B , Fang B . Characteristic expression of apoptotic genes in epulis. Shanghai Kou Qiang Yi Xue. 2020;29(1):31‐35. 32524117

[6] Rohrer MD , Young SK . Congenital epulis (gingival granular cell tumor): ultrastructural evidence of origin from pericytes. Oral Surg Oral Med Oral Pathol. 1982;53(1):56‐63. 6948253 10.1016/0030-4220(82)90486-8

[7] Lazaris AC , Eftychiadis CA , Nicolopoulou P , Paraskevakou HA , Davaris PS . Congenital epulis: an ultrastructural and immunohistochemical case study. Adv Clin Path. 2000;4(4):159‐163. 11436148

[8] Liu J , Wang X , Zheng M , Luan Q . Lipopolysaccharide from Porphyromonas gingivalis promotes autophagy of human gingival fibroblasts through the PI3K/Akt/mTOR signaling pathway. Life Sci. 2018;211:133‐139. 30218719 10.1016/j.lfs.2018.09.023

[9] Zhang Z , Singh R , Aschner M . Methods for the detection of autophagy in mammalian cells. Curr Protoc Toxicol, 2016, 69: 20 12 21‐20 12 26. 27479363 10.1002/cptx.11 PMC4982470

[10] Lowry OH , Rosebrough NJ , Farr AL , Randall RJ . Protein measurement with the folin phenol reagent. J Biol Chem. 1951;193(1):265‐275. 14907713

[11] Doherty J , Baehrecke EH . Life, death and autophagy. Nat Cell Biol. 2018;20(10):1110‐1117. 30224761 10.1038/s41556-018-0201-5 PMC9721133

[12] D'Arcy MS . Cell death: a review of the major forms of apoptosis, necrosis and autophagy. Cell Biol Int. 2019;43(6):582‐592. 30958602 10.1002/cbin.11137

[13] Hengartner MO . The biochemistry of apoptosis. Nature. 2000;407(6805):770‐776. 11048727 10.1038/35037710

[14] Li J , Yuan J . Caspases in apoptosis and beyond. Oncogene. 2008;27(48):6194‐6206. 18931687 10.1038/onc.2008.297

[15] Chao DT , Korsmeyer SJ . BCL‐2 family: regulators of cell death. Annu Rev Immunol. 1998;16:395‐419. 9597135 10.1146/annurev.immunol.16.1.395

[16] Pena‐Blanco A , Garcia‐Saez AJ . Bax, Bak and beyond ‐ mitochondrial performance in apoptosis. FEBS J. 2018;285(3):416‐431. 28755482 10.1111/febs.14186

[17] Fan TJ , Han LH , Cong RS , Liang J . Caspase family proteases and apoptosis. Acta Biochim Biophys Sin (Shanghai). 2005;37(11):719‐727. 16270150 10.1111/j.1745-7270.2005.00108.x

[18] Dikic I , Elazar Z . Mechanism and medical implications of mammalian autophagy. Nat Rev Mol Cell Biol. 2018;19(6):349‐364. 29618831 10.1038/s41580-018-0003-4

[19] Kimmelman AC , White E . Autophagy and tumor metabolism. Cell Metab. 2017;25(5):1037‐1043. 28467923 10.1016/j.cmet.2017.04.004 PMC5604466

[20] Li X , He S , Ma B . Autophagy and autophagy‐related proteins in cancer. Mol Cancer. 2020;19(1):12. 31969156 10.1186/s12943-020-1138-4 PMC6975070

[21] Levy JMM , Towers CG , Thorburn A . Targeting autophagy in cancer. Nat Rev Cancer. 2017;17(9):528‐542. 28751651 10.1038/nrc.2017.53 PMC5975367

[22] Yang X , Yu DD , Yan F , et al. The role of autophagy induced by tumor microenvironment in different cells and stages of cancer. Cell Biosci. 2015;5:14. 25844158 10.1186/s13578-015-0005-2 PMC4384293

[23] Tanida I , Ueno T , Kominami E . LC3 and autophagy. Methods Mol Biol. 2008;445:77‐88. 18425443 10.1007/978-1-59745-157-4_4

[24] Mizushima N . The ATG conjugation systems in autophagy. Curr Opin Cell Biol. 2020;63:1‐10. 31901645 10.1016/j.ceb.2019.12.001

[25] Nishimura T , Tooze SA . Emerging roles of ATG proteins and membrane lipids in autophagosome formation. Cell Discov. 2020;6:32. 10.1038/s41421-020-0161-3 PMC7248066 32509328

[26] Mizushima N , Yoshimori T , Ohsumi Y . The role of Atg proteins in autophagosome formation. Annu Rev Cell Dev Biol. 2011;27:107‐132. 21801009 10.1146/annurev-cellbio-092910-154005

