# Supplementary material for: PCR array analysis identified hyperproliferation but not autophagy or apoptosis in fibrous epulis
Source: J Clin Lab Anal. 2021 May 2;35(6):e23784. doi: 10.1002/jcla.23784 (PMC8183928; doi:10.1002/jcla.23784)
Supplement: Supplementary file 1 — Table S1 [file JCLA-35-e23784-s001.docx]

Supplemental table The result of PCR array

| **Position** | **Symbol** | **Fold Change** | **P-VALUE** |
| --- | --- | --- | --- |
| A01 | AKT1 | 0.947 | 0.659259 |
| A02 | AMBRA1 | 1.4689 | 0.030354 |
| A03 | APP | 1.1262 | 0.519064 |
| A04 | ATG10 | 1.0903 | 0.705668 |
| A05 | ATG12 | 1.1314 | 0.527657 |
| A06 | ATG16L1 | 1.7918 | 0.326354 |
| A07 | ATG16L2 | 0.9084 | 0.287963 |
| A08 | ATG3 | 0.9514 | 0.725027 |
| A09 | ATG4A | 1.1081 | 0.668748 |
| A10 | ATG4B | 1.1904 | 0.100529 |
| A11 | ATG4C | 0.9736 | 0.777469 |
| A12 | ATG4D | 1.0778 | 0.662343 |
| B01 | ATG5 | 1.1472 | 0.553308 |
| B02 | ATG7 | 1.1605 | 0.226579 |
| B03 | ATG9A | 1.134 | 0.401688 |
| B04 | ATG9B | 4.3513 | 0.152388 |
| B05 | BAD | 1.0411 | 0.961245 |
| B06 | BAK1 | 1.103 | 0.548608 |
| B07 | BAX | 1.0704 | 0.672078 |
| B08 | BCL2 | 2.8708 | 0.002084 |
| B09 | BCL2L1 | 2.4894 | 0.007523 |
| B10 | BECN1 | 0.9448 | 0.718083 |
| B11 | BID | 1.0728 | 0.720541 |
| B12 | BNIP3 | 1.001 | 0.937885 |
| C01 | CASP3 | 1.0173 | 0.980452 |
| C02 | CASP8 | 1.1959 | 0.157379 |
| C03 | CDKN1B | 1.7308 | 0.028768 |
| C04 | CDKN2A | 0.9827 | 0.846332 |
| C05 | CLN3 | 1.0556 | 0.612924 |
| C06 | CTSB | 1.022 | 0.899817 |
| C07 | CTSD | 1.3239 | 0.219803 |
| C08 | CTSS | 1.0197 | 0.824163 |
| C09 | CXCR4 | 2.4534 | 0.022578 |
| C10 | DAPK1 | 1.3362 | 0.237594 |
| C11 | DRAM1 | 1.2966 | 0.276232 |
| C12 | DRAM2 | 1.0291 | 0.917745 |
| D01 | EIF2AK3 | 3.2223 | 0.072706 |
| D02 | EIF4G1 | 1.1552 | 0.696357 |
| D03 | ESR1 | 1.6 | 0.197938 |
| D04 | FADD | 0.8301 | 0.352247 |
| D05 | FAS | 0.7569 | 0.152497 |
| D06 | GAA | 1.1959 | 0.258335 |
| D07 | GABARAP | 0.9827 | 0.92678 |
| D08 | GABARAPL1 | 1.8056 | 0.02947 |
| D09 | GABARAPL2 | 0.9872 | 0.974976 |
| D10 | HDAC1 | 0.9405 | 0.765598 |
| D11 | HDAC6 | 1.1822 | 0.240272 |
| D12 | HGS | 1.3208 | 0.231919 |
| E01 | HSP90AA1 | 2.6539 | 0.000496 |
| E02 | HSPA8 | 2.2733 | 0.013304 |
| E03 | HTT | 1.3961 | 0.14225 |
| E04 | IFNG | 1.2467 | 0.415795 |
| E05 | IGF1 | 2.2162 | 0.039901 |
| E06 | INS | 1.2467 | 0.415795 |
| E07 | IRGM | 2.2838 | 0.182405 |
| E08 | LAMP1 | 1.2098 | 0.376611 |
| E09 | MAP1LC3A | 1.9249 | 0.005435 |
| E10 | MAP1LC3B | 3.3053 | 0.101074 |
| E11 | MAPK14 | 1.0173 | 0.958333 |
| E12 | MAPK8 | 1.3961 | 0.106161 |
| F01 | MTOR | 1.0103 | 0.946581 |
| F02 | NFKB1 | 2.1958 | 0.091005 |
| F03 | NPC1 | 1.894 | 0.011101 |
| F04 | PIK3C3 | 1.015 | 0.971232 |
| F05 | PIK3CG | 1.2525 | 0.40535 |
| F06 | PIK3R4 | 0.9895 | 0.846134 |
| F07 | PRKAA1 | 1.0197 | 0.978294 |
| F08 | PTEN | 1.3486 | 0.192565 |
| F09 | RAB24 | 0.958 | 0.70142 |
| F10 | RB1 | 1.1472 | 0.463906 |
| F11 | RGS19 | 1.1552 | 0.676813 |
| F12 | RPS6KB1 | 1.1158 | 0.516109 |
| G01 | SNCA | 7.8977 | 0.309389 |
| G02 | SQSTM1 | 1.2877 | 0.022928 |
| G03 | TGFB1 | 1.0828 | 0.793993 |
| G04 | TGM2 | 1.4963 | 0.174763 |
| G05 | TMEM74 | 1.3057 | 0.283517 |
| G06 | TNF | 1.6481 | 0.036958 |
| G07 | TNFSF10 | 2.0822 | 0.294238 |
| G08 | TP53 | 0.9895 | 0.882465 |
| G09 | ULK1 | 1.5243 | 0.188524 |
| G10 | ULK2 | 1.979 | 0.0259 |
| G11 | UVRAG | 1.6299 | 0.009688 |
| G12 | WIPI1 | 1.2438 | 0.173677 |
| H01 | ACTB | 0.9232 | 0.880682 |
| H02 | B2M | 1.0126 | 0.852332 |
| H03 | GAPDH | 1.0056 | 0.915105 |
| H04 | HPRT1 | 0.9804 | 0.771165 |
| H05 | RPLP0 | 0.9827 | 0.794387 |
| H06 | HGDC | 1.2467 | 0.415795 |
| H07 | RTC | 1.0532 | 0.709045 |
| H08 | RTC | 1.0828 | 0.598153 |
| H09 | RTC | 1.1659 | 0.361001 |
| H10 | PPC | 1.174 | 0.447196 |
| H11 | PPC | 1.1931 | 0.383967 |
| H12 | PPC | 0.8674 | 0.554855 |
